# Absolute configuration of (2*S*)-4-(4-hy­droxy­phen­yl)butan-2-ol

**DOI:** 10.1107/S1600536811010026

**Published:** 2011-03-23

**Authors:** S. Yousuf, S. G. Musharraf, I. Khan, Samiullah, Hoong-Kun Fun

**Affiliations:** a H.E.J. Research Institute of Chemistry, International Center for Chemical and Biological Sciences, University of Karachi 75270, Pakistan; b Department of Pharmacy, University of Peshawar, Peshawar 25120, Pakistan; c X-ray Crystallography Unit, School of Physics, Universiti Sains Malaysia, 11800 USM, Penang, Malaysia

## Abstract

The title compound, C_10_H_14_O_2_, was isolated from the chloro­form extract of *Taxus wallichiana* Zucc. In the crystal, mol­ecules are linked by inter­molecular O—H⋯O hydrogen bonds, forming sheets parallel to (100). There are weak inter­molecular C—H⋯π inter­actions between the sheets.

## Related literature

For the isolation of the title compound, see: Fan *et al.* (1999 ▶). For the biological activity and medicinal uses of *Taxus. wallichiana* Zucc, see: Ahmed (1997 ▶); Baquar (1995 ▶); Kaul (1997 ▶); Nisar *et al.* (2008*a* ▶,*b* ▶; 2010 ▶); Prasain *et al.* (2001 ▶); Wani *et al.* (1971 ▶).
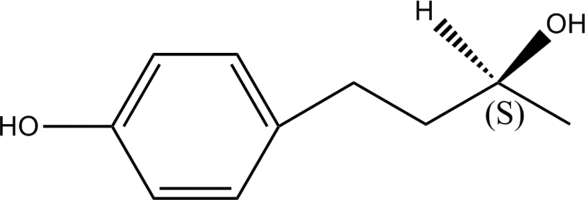


## Experimental

### 

#### Crystal data

C_10_H_14_O_2_
*M* *_r_* = 166.21
Monoclinic, 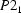

*a* = 7.2342 (2) Å
*b* = 6.3815 (2) Å
*c* = 9.9419 (4) Å
β = 92.216 (2)°
*V* = 458.63 (3) Å^3^
*Z* = 2
Cu *K*α radiation
μ = 0.66 mm^−1^
*T* = 100 K
0.39 × 0.12 × 0.05 mm

#### Data collection

Bruker SMART APEXII DUO CCD area-detector diffractometer
Absorption correction: multi-scan (*SADABS*; Bruker, 2009 ▶) *T*_min_ = 0.784, *T*_max_ = 0.966
4954 measured reflections
1455 independent reflections
1446 reflections with *I* > 2σ(*I*)
*R*_int_ = 0.028

#### Refinement

*R*[*F* ^2^ > 2σ(*F* ^2^)] = 0.029
*wR*(*F* ^2^) = 0.075
*S* = 1.08
1455 reflections
165 parameters
1 restraint
H atoms treated by a mixture of independent and constrained refinement
Δρ_max_ = 0.19 e Å^−3^
Δρ_min_ = −0.24 e Å^−3^
Absolute structure: Flack (1983 ▶), 579 Friedel pairs
Flack parameter: −0.03 (17)

### 

Data collection: *APEX2* (Bruker, 2009 ▶); cell refinement: *SAINT* (Bruker, 2009 ▶); data reduction: *SAINT*; program(s) used to solve structure: *SHELXS97* (Sheldrick, 2008 ▶); program(s) used to refine structure: *SHELXL97* (Sheldrick, 2008 ▶); molecular graphics: *SHELXTL* (Sheldrick, 2008 ▶) and *PLATON* (Spek, 2009 ▶); software used to prepare material for publication: *SHELXTL* and *PLATON*.

## Supplementary Material

Crystal structure: contains datablocks global, I. DOI: 10.1107/S1600536811010026/lh5221sup1.cif

Structure factors: contains datablocks I. DOI: 10.1107/S1600536811010026/lh5221Isup2.hkl

Additional supplementary materials:  crystallographic information; 3D view; checkCIF report

## Figures and Tables

**Table 1:** Hydrogen-bond geometry (Å, °) *Cg*1 is the centroid of the C1–C6 ring

*D*—H⋯*A*	*D*—H	H⋯*A*	*D*⋯*A*	*D*—H⋯*A*
O1—H1*O*1⋯O2^i^	0.92 (2)	1.72 (2)	2.6247 (13)	166 (2)
O2—H1*O*2⋯O1^ii^	0.91 (2)	1.88 (2)	2.7869 (13)	177.8 (19)
C8—H8*A*⋯*Cg*1^iii^	0.93 (2)	2.822 (15)	3.7033 (13)	158.1 (14)

Symmetry codes: (i) 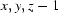; (ii) 
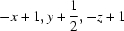
; (iii) 
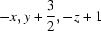
.

**Fig. 1.**
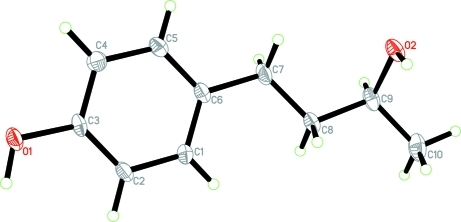
The molecular structure of the title compound, showing 50% probability displacement ellipsoids.

**Fig. 2.**
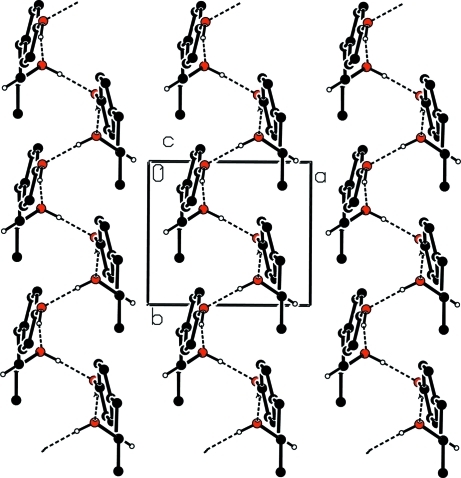
Part of the crystal structure with O-H···O hydrogen bonds shown as dashed lines. Only H atoms involved in hydrogen bonds are included.

## supplementary crystallographic information

### Comment

The genus *Taxus* belonging to the family *Taxaceae* is well known for its anticancer agents, namely taxol and dosetaxel (Wani *et al.*, 1971; Prasain *et al.*, 2001; Nisar *et al.*, 2008*a*,*b*;2010). *Taxus. wallichiana* Zucc. (Himalayan Yew) is a small to medium sized evergreen tree, native to the northern areas of the Pakistan. Literature survey revealed that this plant is used traditionally for the treatment of high fever and acute painful conditions (Kaul, 1997). Leaves of the plant are used to make herbal tea for indigestion and epilepsy (Baquar, 1995; Ahmed, 1997). During our ongoing search on the medicinally important plants of Pakistan, the title compound was isolated from the chloroform-soluble part of *Taxus. wallichiana* and the structure was established on the basis of X-ray diffraction studies.

The molecular structure of the title compound is shown in Fig. 1. In the crystal structure, molecules are linked by intermolecular O1—H1O1···O2^i^ and O2—H1O2···O1^ii^ (see Table 1 for symmetry codes) hydrogen bonds to form two-dimensional sheets paralel to the (100) (Fig.2). A weak C—H···*π* interaction is also observed.

### Experimental

Plant material:*Taxus. wallichiana* Zucc. was collected from the Hazara division of the North-western Frontier Province, Pakistan, in March 2005 and identified by Dr. Hasan Sher, a taxonomist of the Department of Botany, Jehanzeb Postgraduate College Saidu Sharif, Swat, Pakistan. A voucher specimen was deposited in the herbarium of the same institution. The aerial parts of the plant were air-dried under shade for six consecutive weeks at room temperature. The dried plant material was later on chopped, finely ground and stored in polyethylene bags under refrigeration for further experimentation.

The isolation of 4-(4'-hydroxyphenyl)-(2S)-butanol was previously carried out by Fan *et al.* (1999). Extraction and purification: The air-dried and powdered bark (4.0 K g) was macerated in methanol with occasional manual shaking at room temperature for a period of 72 h. The process was repeated 3 times followed by filtration. The combined filtrates were evaporated under reduced pressure at 313K to obtain a crude gummy material (514 g, 12.85% *w*/*w*), which was suspended in distilled water and successively extracted with hexane (11% *w*/*w*), chloroform (31.9% *w*/*w*), ethyl acetate (38.8% *w*/*w*), and finally with water (18.2% *w*/*w*) to give the respective fraction. The chloroform fraction (182 g) was further separated using silica gel column chromatography (95 mm in diameter). The column was eluted with increasing polarities of *n*-hexane with chloroform (1%-100%) followed by the gradient mixtures of chloroform and methanol. The methanol gradient was increased carefully to collect twenty seven sub-fractions (C1—C27). The sub-fraction C17 (611 mg) obtained on elution with 6% methanol/chloroform mixture was finally purified by flash column chromatography (silica gel, 8 g) by using chloroform: methanol; (95:5) as eluent to yield a colorless crystalline compound, 4-(4'-hydroxyphenyl)-(2*S*)-butanol (928 mg). Crystals suitable for X-ray diffraction were grown from a solution of the title compound in a mixture of chloroform: methanol (95:5).

### Refinement

The hydrogen atoms were located in difference Fourier maps and refined isotropically. The C—H distances refined in the range 0.94 (2)-1.02 (2)Å.

### Crystal data

**Table:**

C_10_H_14_O_2_	*F*(000) = 180
*M**_r_* = 166.21	*D*_x_ = 1.204 Mg m^−^^3^
Monoclinic, *P*2_1_	Cu *K*α radiation, λ = 1.54178 Å
Hall symbol: P 2yb	Cell parameters from 3477 reflections
*a* = 7.2342 (2) Å	θ = 6.1–70.3°
*b* = 6.3815 (2) Å	µ = 0.66 mm^−^^1^
*c* = 9.9419 (4) Å	*T* = 100 K
β = 92.216 (2)°	Block, colorles
*V* = 458.63 (3) Å^3^	0.39 × 0.12 × 0.05 mm
*Z* = 2	

### Data collection

**Table:**

Bruker SMART APEXII DUO CCD area-detector diffractometer	1455 independent reflections
Radiation source: fine-focus sealed tube	1446 reflections with *I* > 2σ(*I*)
graphite	*R*_int_ = 0.028
φ and ω scans	θ_max_ = 67.5°, θ_min_ = 6.1°
Absorption correction: multi-scan (*SADABS*; Bruker, 2009)	*h* = −8→8
*T*_min_ = 0.784, *T*_max_ = 0.966	*k* = −7→7
4954 measured reflections	*l* = −11→10

### Refinement

**Table:**

Refinement on *F*^2^	Secondary atom site location: difference Fourier map
Least-squares matrix: full	Hydrogen site location: inferred from neighbouring sites
*R*[*F*^2^ > 2σ(*F*^2^)] = 0.029	H atoms treated by a mixture of independent and constrained refinement
*wR*(*F*^2^) = 0.075	*w* = 1/[σ^2^(*F*_o_^2^) + (0.0499*P*)^2^ + 0.0461*P*] where *P* = (*F*_o_^2^ + 2*F*_c_^2^)/3
*S* = 1.08	(Δ/σ)_max_ = 0.001
1455 reflections	Δρ_max_ = 0.19 e Å^−^^3^
165 parameters	Δρ_min_ = −0.23 e Å^−^^3^
1 restraint	Absolute structure: Flack (1983), 579 Friedel pairs
Primary atom site location: structure-invariant direct methods	Flack parameter: −0.03 (17)

### Special details

**Table:**

Experimental. The crystal was placed in the cold stream of an Oxford Cryosystems Cobra open-flow nitrogen cryostat (Cosier & Glazer, 1986) operating at 100.0 (1) K. Cosier, J. & Glazer, A. M. (1986). *J. Appl. Cryst.*19, 105–107.
Geometry. All e.s.d.'s (except the e.s.d. in the dihedral angle between two l.s. planes) are estimated using the full covariance matrix. The cell e.s.d.'s are taken into account individually in the estimation of e.s.d.'s in distances, angles and torsion angles; correlations between e.s.d.'s in cell parameters are only used when they are defined by crystal symmetry. An approximate (isotropic) treatment of cell e.s.d.'s is used for estimating e.s.d.'s involving l.s. planes.
Refinement. Refinement of *F*^2^ against ALL reflections. The weighted *R*-factor *wR* and goodness of fit *S* are based on *F*^2^, conventional *R*-factors *R* are based on *F*, with *F* set to zero for negative *F*^2^. The threshold expression of *F*^2^ > σ(*F*^2^) is used only for calculating *R*-factors(gt) *etc*. and is not relevant to the choice of reflections for refinement. *R*-factors based on *F*^2^ are statistically about twice as large as those based on *F*, and *R*- factors based on ALL data will be even larger.

### Fractional atomic coordinates and isotropic or equivalent isotropic displacement parameters (Å^2^)

**Table:**

	*x*	*y*	*z*	*U*_iso_*/*U*_eq_	
O1	0.33418 (12)	0.03888 (15)	0.11761 (9)	0.0200 (2)	
O2	0.33463 (12)	0.33901 (17)	0.93717 (9)	0.0214 (2)	
C1	0.22229 (17)	0.3267 (2)	0.42530 (13)	0.0191 (3)	
C2	0.25737 (17)	0.2886 (2)	0.29010 (14)	0.0184 (3)	
C3	0.29911 (15)	0.08712 (19)	0.24875 (13)	0.0161 (3)	
C4	0.30875 (17)	−0.0757 (2)	0.34234 (14)	0.0191 (3)	
C5	0.27631 (16)	−0.0347 (2)	0.47664 (13)	0.0187 (3)	
C6	0.23165 (16)	0.1659 (2)	0.52052 (13)	0.0173 (3)	
C7	0.19572 (19)	0.2007 (2)	0.66828 (14)	0.0222 (3)	
C8	0.20129 (16)	0.4288 (2)	0.71453 (13)	0.0179 (3)	
C9	0.18659 (17)	0.4510 (2)	0.86638 (13)	0.0202 (3)	
C10	0.1875 (2)	0.6786 (3)	0.91038 (16)	0.0286 (4)	
H1	0.192 (2)	0.463 (3)	0.4534 (16)	0.019 (4)*	
H2	0.245 (2)	0.401 (3)	0.2254 (19)	0.026 (4)*	
H4	0.339 (2)	−0.216 (3)	0.3145 (17)	0.024 (4)*	
H5	0.281 (2)	−0.143 (3)	0.5411 (18)	0.025 (4)*	
H7A	0.076 (2)	0.136 (3)	0.6896 (18)	0.027 (4)*	
H7B	0.293 (3)	0.123 (3)	0.720 (2)	0.032 (5)*	
H8A	0.103 (3)	0.501 (3)	0.6728 (18)	0.026 (4)*	
H8B	0.322 (2)	0.494 (3)	0.6865 (15)	0.013 (4)*	
H9	0.078 (2)	0.383 (3)	0.8913 (17)	0.020 (4)*	
H10A	0.075 (3)	0.755 (4)	0.873 (2)	0.044 (6)*	
H10B	0.304 (2)	0.749 (3)	0.8792 (17)	0.021 (4)*	
H10C	0.183 (3)	0.691 (3)	1.011 (2)	0.040 (5)*	
H1O1	0.319 (3)	0.151 (4)	0.062 (2)	0.043 (6)*	
H1O2	0.444 (3)	0.400 (3)	0.920 (2)	0.036 (5)*	

### Atomic displacement parameters (Å^2^)

**Table:**

	*U*^11^	*U*^22^	*U*^33^	*U*^12^	*U*^13^	*U*^23^
O1	0.0240 (4)	0.0247 (5)	0.0115 (5)	0.0004 (4)	0.0046 (3)	−0.0002 (4)
O2	0.0213 (5)	0.0303 (5)	0.0125 (5)	−0.0034 (4)	0.0007 (3)	0.0033 (4)
C1	0.0245 (6)	0.0192 (7)	0.0135 (6)	−0.0005 (5)	0.0016 (5)	−0.0021 (5)
C2	0.0220 (6)	0.0222 (8)	0.0110 (6)	−0.0008 (5)	0.0003 (5)	0.0023 (5)
C3	0.0138 (6)	0.0244 (7)	0.0101 (6)	−0.0007 (5)	0.0023 (4)	−0.0021 (5)
C4	0.0188 (6)	0.0195 (7)	0.0193 (7)	0.0006 (5)	0.0038 (4)	0.0005 (5)
C5	0.0201 (6)	0.0218 (7)	0.0142 (7)	−0.0017 (5)	0.0018 (4)	0.0029 (5)
C6	0.0180 (6)	0.0223 (7)	0.0115 (7)	−0.0027 (5)	0.0003 (5)	0.0003 (5)
C7	0.0304 (7)	0.0262 (8)	0.0102 (7)	−0.0047 (6)	0.0024 (5)	−0.0003 (5)
C8	0.0172 (6)	0.0247 (7)	0.0119 (6)	−0.0009 (5)	0.0004 (4)	0.0005 (5)
C9	0.0161 (6)	0.0325 (8)	0.0123 (7)	−0.0010 (5)	0.0024 (4)	−0.0019 (6)
C10	0.0278 (7)	0.0378 (9)	0.0201 (8)	0.0062 (7)	−0.0012 (5)	−0.0108 (6)

### Geometric parameters (Å, °)

**Table:**

O1—C3	1.3728 (15)	C5—H5	0.94 (2)
O1—H1O1	0.91 (3)	C6—C7	1.5180 (17)
O2—C9	1.4473 (16)	C7—C8	1.5265 (19)
O2—H1O2	0.90 (2)	C7—H7A	0.988 (19)
C1—C6	1.3959 (19)	C7—H7B	0.99 (2)
C1—C2	1.3988 (18)	C8—C9	1.5241 (17)
C1—H1	0.943 (19)	C8—H8A	0.93 (2)
C2—C3	1.3865 (18)	C8—H8B	1.018 (15)
C2—H2	0.96 (2)	C9—C10	1.517 (2)
C3—C4	1.3948 (18)	C9—H9	0.938 (17)
C4—C5	1.390 (2)	C10—H10A	1.01 (2)
C4—H4	0.97 (2)	C10—H10B	1.013 (18)
C5—C6	1.3942 (19)	C10—H10C	1.01 (2)
			
C3—O1—H1O1	112.1 (15)	C8—C7—H7A	110.2 (11)
C9—O2—H1O2	109.6 (13)	C6—C7—H7B	106.5 (12)
C6—C1—C2	121.21 (12)	C8—C7—H7B	108.1 (12)
C6—C1—H1	118.9 (10)	H7A—C7—H7B	107.0 (15)
C2—C1—H1	119.9 (10)	C9—C8—C7	112.65 (11)
C3—C2—C1	119.72 (12)	C9—C8—H8A	108.3 (11)
C3—C2—H2	120.5 (11)	C7—C8—H8A	109.0 (12)
C1—C2—H2	119.7 (11)	C9—C8—H8B	109.0 (9)
O1—C3—C2	122.67 (11)	C7—C8—H8B	108.8 (9)
O1—C3—C4	117.40 (11)	H8A—C8—H8B	109.0 (14)
C2—C3—C4	119.93 (11)	O2—C9—C10	109.73 (11)
C5—C4—C3	119.64 (12)	O2—C9—C8	110.92 (10)
C5—C4—H4	119.9 (11)	C10—C9—C8	112.02 (12)
C3—C4—H4	120.4 (11)	O2—C9—H9	104.8 (11)
C4—C5—C6	121.57 (12)	C10—C9—H9	111.0 (11)
C4—C5—H5	120.8 (12)	C8—C9—H9	108.0 (10)
C6—C5—H5	117.6 (12)	C9—C10—H10A	111.3 (13)
C5—C6—C1	117.92 (12)	C9—C10—H10B	109.5 (11)
C5—C6—C7	119.18 (11)	H10A—C10—H10B	109.7 (16)
C1—C6—C7	122.90 (12)	C9—C10—H10C	111.3 (13)
C6—C7—C8	115.31 (11)	H10A—C10—H10C	105.7 (17)
C6—C7—H7A	109.3 (11)	H10B—C10—H10C	109.3 (15)
			
C6—C1—C2—C3	1.14 (18)	C2—C1—C6—C5	−0.29 (18)
C1—C2—C3—O1	179.72 (10)	C2—C1—C6—C7	179.64 (11)
C1—C2—C3—C4	−0.99 (17)	C5—C6—C7—C8	163.67 (11)
O1—C3—C4—C5	179.33 (10)	C1—C6—C7—C8	−16.26 (18)
C2—C3—C4—C5	0.01 (17)	C6—C7—C8—C9	−173.32 (10)
C3—C4—C5—C6	0.86 (17)	C7—C8—C9—O2	58.43 (14)
C4—C5—C6—C1	−0.71 (17)	C7—C8—C9—C10	−178.58 (11)
C4—C5—C6—C7	179.36 (11)		

### Hydrogen-bond geometry (Å, °)

**Table:**

Cg1 is the centroid of the C1–C6 ring

**Table:**

*D*—H···*A*	*D*—H	H···*A*	*D*···*A*	*D*—H···*A*
O1—H1O1···O2^i^	0.92 (2)	1.72 (2)	2.6247 (13)	166 (2)
O2—H1O2···O1^ii^	0.91 (2)	1.88 (2)	2.7869 (13)	177.8 (19)
C8—H8A···Cg1^iii^	0.93 (2)	2.822 (15)	3.7033 (13)	158.1 (14)

Symmetry codes: (i) *x*, *y*, *z*−1; (ii) −*x*+1, *y*+1/2, −*z*+1; (iii) −*x*, *y*+3/2, −*z*+1.

## References

[bb1] Ahmed, B. (1997). *J. Hamdard Med.* **20**, 53–54.

[bb2] Baquar, S. R. (1995). *Trees of Pakistan: Their Natural History Characteristics and Utilization*, p. 634. Karachi: Royal Book Company.

[bb3] Bruker (2009). *APEX2*, *SAINT* and *SADABS* Bruker AXS Inc., Madison, Wisconsin, USA.

[bb4] Fan, C. Q., Yang, G. J., Zhao, W. M., Ding, B. Y. & Qin, Q. W. (1999). *Chin. Chem. Lett.* **10**, 567–570.

[bb5] Flack, H. D. (1983). *Acta Cryst.* A**39**, 876–881.

[bb6] Kaul, M. K. (1997). *Medicinal Plants of Kashmir and Ladakh: Temperate and Cold Arid Himalaya*, p. 173. New Delhi, India: Indus Publishing Company.

[bb7] Nisar, M., Khan, I., Ali, I., Ahmad, W. & Choudhary, M. I. (2008*a*). *J. Enz. Inhib. Med. Chem.* **23**, 256–260. 10.1080/14756360701505336 18343912

[bb8] Nisar, M. Khan, I., Simjee, S. U., Gilani, A. H. Obaidullah, Perveen, H., (2008*b*). *J. Ethnopharm.* **116**, 490–494. 10.1016/j.jep.2007.12.021 18308491

[bb9] Nisar, M., Qayum, M., Adhikari, A., Khan, I., Kaleem, A. K., Ali, Z. & Choudhary, M. I. (2010). *Nat. Prod. Commun.* **5**, 1727–1728. 21213967

[bb10] Prasain, J. K., Stefanowicz, P., Kiyota, T., Habeichi, F. & Konishi, Y. (2001). *Phytochemistry*, **58**, 1167–1170. 10.1016/s0031-9422(01)00305-3 11738401

[bb11] Sheldrick, G. M. (2008). *Acta Cryst.* A**64**, 112–122. 10.1107/S0108767307043930 18156677

[bb12] Spek, A. L. (2009). *Acta Cryst.* D**65**, 148–155. 10.1107/S090744490804362X PMC2631630 19171970

[bb13] Wani, M. C., Taylor, H. L., Wall, M. E., Coggon, P. & McPhail, A. T. (1971). *J. Am. Chem. Soc.* **93**, 2325–2327. 10.1021/ja00738a045 5553076

